# Male eyespan size is associated with meiotic drive in wild stalk-eyed flies (*Teleopsis dalmanni*)

**DOI:** 10.1038/hdy.2013.131

**Published:** 2014-01-08

**Authors:** A J Cotton, M Földvári, S Cotton, A Pomiankowski

**Affiliations:** 1 Department of Genetics, Evolution and Environment, University College London, London, UK; 2 CoMPLEX, University College London, London, UK; 3 MTA-DE ‘Lendület' Behavioural Ecology Research Group, Department of Evolutionary Zoology, University of Debrecen, 1, Egyetem tér, Debrecen, Hungary

**Keywords:** meiotic drive, sex ratio, sexual selection, stalk-eyed fly

## Abstract

This study provides the first direct evidence from wild populations of stalk-eyed flies to support the hypothesis that male eyespan is a signal of meiotic drive. Several stalk-eyed fly species are known to exhibit X-linked meiotic drive. A recent quantitative trait locus analysis in *Teleopsis dalmanni* found a potential link between variation in male eyespan, a sexually selected ornamental trait, and the presence of meiotic drive. This was based on laboratory populations subject to artificial selection for male eyespan. In this study, we examined the association between microsatellite markers and levels of sex ratio bias (meiotic drive) in 12 wild *T. dalmanni* populations. We collected two data sets: (a) brood sex ratios of wild-caught males mated to standard laboratory females and (b) variation in a range of phenotypic traits associated with reproductive success of wild-caught males and females. In each case, we typed individuals for eight X-linked microsatellite markers, including several that previously were shown to be associated with male eyespan and meiotic drive. We found that one microsatellite marker was very strongly associated with meiotic drive, whereas a second showed a weaker association. We also found that, using both independent data sets, meiotic drive was strongly associated with male eyespan, with smaller eyespan males being associated with more female-biased broods. These results suggest that mate preference for exaggerated male eyespan allows females to avoid mating with males carrying the meiotic drive gene and is thus a potential mechanism for the maintenance and evolution of female mate preference.

## Introduction

The majority of species have ∼1:1 offspring sex ratios. The prevalence of this phenomenon has been explained by adaptive sex ratio theory (Fisher, 1930). If one sex were to become increasingly rare in the population, then selection would favour individuals that produced the rarer sex, thereby returning the overall population to a 1:1 sex ratio (Fisher, 1930). A number of forces, including local mate competition and differential payoffs for the sexes against environmental gradients, can lead to well-characterised deviations from a balanced sex ratio (Hamilton, 1967).

However, deviations from 1:1 ratios can also be caused by a range of selfish genetic elements that promote their own transmission to the next generation, at the expense of the rest of the genome. Selfish genetic elements further their interests in ways that result in the distortion of the normal offspring sex ratio. Examples are widespread in eukaryotes, with a range of tactics used by different types of selfish genetic elements (Hurst and Werren, 2001). One common form of selfish genetic element is sex chromosome meiotic drive, usually linked to the X chromosome and active in the heterogametic sex in species with the XY sex-determination system (Hurst and Pomiankowski, 1991; Lyttle, 1993). Individuals that possess the driving X chromosome (X^D^) produce female-biased offspring sex ratios (Hamilton, 1967). This is typically due to differential sperm maturation or survival during spermatogenesis (Lyttle, 1993). The sperm of a number of species fail to undergo complete spermatid development and individualisation, leading to low survival among Y-bearing sperm and few male offspring, for example, in *Drosophila melanogaster* (Tokuyasu *et al.*, 1972), *Drosophila simulans* (Montchamp-Moreau and Joly, 1997; Cazemajor *et al.*, 2000) and *Teleopsis whitei* (formerly *Cyrtodiopsis whitei*) (Wilkinson and Sanchez, 2001).

All studies to date have found that meiotic drive systems require at least two distinct linked loci, a drive and its target or responder (Lyttle, 1993; Larracuente and Presgraves, 2012). Associated inversions limit recombination allowing the drive and responder loci to remain in tight linkage (Wu and Beckenbach, 1983). Only a small number of meiotic drive systems have been studied in detail, the best known being the *t*-complex in mice (Silver, 1993), the segregation distortion (*Sd*) system in *D. melanogaster* (Kusano *et al.*, 2003) and the *sex-ratio* system in *D. simulans* (Cazemajor *et al.*, 2000). Given that the ramifications of meiotic drive can range from intragenomic conflict to species-level extinction (Jaenike, 2001), there is a great need to study the selective and ecological processes that are involved in the evolution and maintenance of meiotic drive in wild populations. Here we examine the meiotic drive system in the stalk-eyed fly *T. dalmanni* and relate the pattern of drive to the operation of sexual selection in wild populations.

Stalk-eyed flies display a unique form of hypercephaly whereby the head capsule is elongated in the form of eyestalks, causing the lateral displacement of the eyes to the end of these stalks. Although many families in the order Diptera exhibit this type of hypercephaly, the diopsid family is distinctive in that both sexes in all species display this trait (Wilkinson and Dodson, 1997). Many species of this family exhibit sexual dimorphism of eyespan (the distance between the outer most edge of the eyes), with males possessing a significantly larger eyespan, relative to their body size, than females (Burkhardt and de la Motte, 1985). Numerous studies have shown that exaggerated male eyespan has evolved through sexual selection, with the trait used in mate choice (Wilkinson and Reillo, 1994; Cotton *et al.*, 2010) and male antagonistic interactions (Small *et al.*, 2009).

One of the most intensively studied stalk-eyed flies is the Malaysian species, *Teleopsis dalmanni*. Both sexes spend their day foraging independently on decaying plant matter, and at dusk they congregate on exposed root hairs overhanging the eroded banks of rainforest streams (Wilkinson, 1993; Wilkinson and Reillo, 1994; Cotton *et al.*, 2010). Females choose their roosting sites (and therefore mates) from among the root hairs where males have established themselves, resulting in a ‘lek' style mating system (Cotton *et al.*, 2010). Males aggressively compete with each other for control of these sites (Small *et al.*, 2009) and females prefer to roost and mate with males with larger (absolute and relative) eyespan (Wilkinson and Reillo, 1994; Cotton *et al.*, 2010). A variety of laboratory studies have provided key data on reproductive traits in males and females. In the laboratory, male accessory gland size co-varies with male mating frequency, both phenotypically (Rogers *et al.*, 2005) and genetically (Baker *et al.*, 2003). Accessory glands become depleted with repeated matings (Rogers *et al.*, 2006), and the amount of sperm stored by a female is correlated to the testis size of the male that she mates with (Fry, 2006). A similar pattern of co-variation between male eyespan and the size of the testes and the accessory glands has been found in the wild (Cotton *et al.*, 2010).

Presgraves *et al.* (1997) first reported the existence of sex chromosome meiotic drive in two *Teleopsis* species (*T. dalmanni* and *T. whitei*). In the laboratory, genetic analyses revealed that both species had high levels of female-biased broods (13–17% and 29%, respectively) and that the sex ratio bias was caused by spermatid degeneration in X^D^ males, similar to that seen in a number of *Drosophila* species (for example, Montchamp-Moreau and Joly, 1997). Wilkinson *et al.* (1998) proposed that females might benefit by choosing males that are resistant to meiotic drive in order to gain by producing more male offspring. To test this, they artificially selected male flies for relatively large and relatively small eyespan for 22 generations and found a correlation between eyespan and offspring sex ratios. In one of the pair of replicated small eyespan lines there was a bias towards female-biased broods, whereas both large eyespan lines produced fewer female-biased broods. These results suggest that drive may be associated with reduced sexual signalling, and that male eyespan is subject to a form of ‘good genes' sexual selection through mate preference for drive resistance (Wilkinson *et al.*, 1998).

To take this analysis further, Johns *et al.* (2005) investigated linkage patterns between microsatellite loci associated with meiotic drive and eyespan. They crossed two of the artificially selected lines (small × large) that showed significantly biased sex ratios, genotyped F2 individuals and found an X^D^-specific haplotype consisting of four X-linked microsatellite markers (*ms54*, *ms125*, *ms244* and *ms395*). The linkage analysis revealed a dramatic reduction in recombination between the X^D^ and the standard non-drive X chromosome, indicative that X^D^ is located in a region of low recombination (for example, an inversion). An X-linked quantitative trait locus, which explained 36% of the variation in male eyespan, was found to be located only 1.3 cM from the drive locus on the X chromosome, suggesting a close physical association between a major locus for eyespan and the locus for drive (Johns *et al.*, 2005). This work again suggests that there is an association between meiotic drive and male eyespan. However, given that only two artificially selected lines were manipulated, it is plausible that the observed genetic linkage could simply be due to chance. A more extensive analysis is needed to establish the strength of the association and the predictive power of the microsatellites investigated.

The work to date investigating meiotic drive in stalk-eyed flies was carried out on laboratory populations. There remains little knowledge of either the frequency or distribution of meiotic drive in natural populations of *T. dalmanni*. In addition, despite the potential importance of the hypothesis linking male signalling with meiotic drive, this association has not been tested against data from populations in the wild. To address this, we analysed whether the microsatellites previously linked with meiotic drive in laboratory studies showed the same pattern in natural populations. Using a large sample of male and female flies from 12 wild populations, we examined natural levels of microsatellite variation. We then looked for associations between male eyespan and meiotic drive directly, as well as with those microsatellite loci that had been putatively linked to meiotic drive. In addition, we tested whether these microsatellites were associated with traits that predict reproductive success in males (testis and accessory gland size) and in females (fecundity).

## Materials and methods

### Source of experimental flies

#### Wild flies

**Figure 1:**
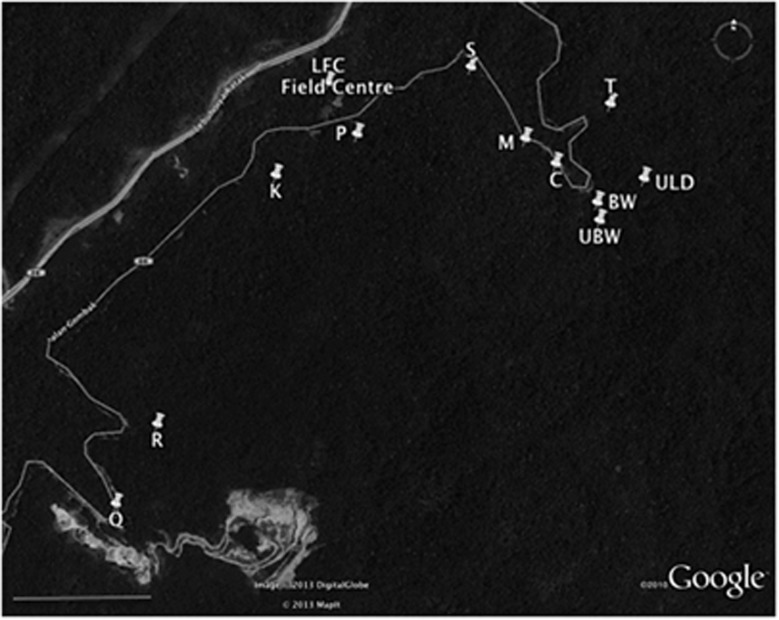
Map showing the 12 sites used for collections and the University of Malaya Field Studies Centre. All sites represent distinct populations that lie along or near to the small Gombak road, Jalan Gombak, which runs through mountainous rainforest. To the upper left is a major motorway in the valley. In addition to the rainforest, the map also shows the local quarry (bottom centre left). A compass is shown for orientation and the bar on the bottom left indicates a scale of 1000 m. Google Earth Image © 2013 DigitalGlobe © 2013 MapIt.

All analyses were carried out using flies collected from 12 sites along the Ulu Gombak valley, in Peninsular Malaysia, spanning ∼5 km. The sites were as follows: Blair Witch (BW) (3°19′N 101°45′E); Cascade (C) (3°19′N 101°45′E); Kingfisher (K) (3°19′N 101°45′E); Lower Field Centre (LFC) (3°19′N 101°45′E); Mihaly (M) (3°19′N 101°45′E); Poppet (P) (3°19′N 101°45′E); Quarry (Q) (3°18′N 101°44′E); Rubbish (R) (3°18′N 101°44′E); Swamp (S) (3°19′N 101°45′E); Tarantula (T) (3°19′N 101°45′E); Upper Blair Witch (UBW) (3°19′N 101°45′E); and Upper Lazy Dog (ULD) (3°19′N 101°45′E) (Figure 1). These sites are a mix of primary and secondary rainforest, 20–40 m in length (along a stream), with rootlets found under hanging stream banks.

#### Laboratory stock

A large sample of *T. dalmanni* was collected in 2005 (by SC and AP), from the Ulu Gombak valley, Peninsular Malaysia (3°19′N 101°45′E). All flies (both laboratory and experimental) were collected at night with small clear plastic bags placed over the rootlet trapping the flies inside. This allowed the gentle removal of the whole ‘lek' in clearly labelled individual bags. These were then transferred into pots at the field centre. Since transportation back to the UK, flies have been maintained in cage culture at high density (>200 individuals) with an ∼1:1 sex ratio to minimise inbreeding. The population was kept at 25 °C, with a 12:12 h dark: light cycle and fed pureed sweet corn twice weekly.

### Wild males

Male flies (*N=*134) were collected from five sites (BW, C, Q, UBW and ULD) in September 2009 (*N*=31) and September 2011 (*N*=103). They were transported to the UK, individually housed in 400 ml pots, fed on pureed sweet corn twice a week and kept in constant temperature rooms at 25 °C on a 12:12 h light: dark cycle. Three virgin laboratory females were added to each male pot. Flies were allowed to mate freely. The bases of the pots were lined with a moist cotton pad and blue paper to allow for easy egg visualisation. Eggs were collected twice a week for 3 weeks and kept in Petri dishes lined with a moist cotton pad. Pupae were allowed to eclose into cage culture, and the resulting flies (offspring) were counted and sexed, and an offspring sex ratio was assigned to each male (see below). Male flies were anaesthetised on ice and stored in 100% ethanol.

### Adult phenotypes

Adult male (*N=*226) and female (*N=*210) flies were collected from all 12 sites along the Ulu Gombak valley in August 2008. Flies were anaesthetised on ice shortly after capture and digital images taken using a monocular field microscope in order to measure eyespan (the distance between the outer edges of the eye bulbs) and thorax length (the distance from the base of the head to the posterior edge of the thorax and is measured as a proxy for body size) to an accuracy of 0.01 mm, using NIH Image software (v. 1.55, National Institutes of Health, Bethesda, MD, USA). The reproductive tract of each female was dissected and fecundity was measured as the number of mature eggs in the ovaries. The reproductive tract of each male was dissected into phosphate saline buffer. The accessory glands and testis were extracted and uncoiled, placed on a graticule and photographed digitally under a monocular field microscope (Baker *et al.*, 2003). The length of both the testis and accessory glands were then measured. All of these flies were stored in 100% ethanol.

The density of flies at each of the 12 sample sites was calculated using an average based on three collections taken at the same sample sites over 3 years (August 2008, March 2009 and September 2010). The density was estimated as the number of flies collected per metre of site sampled.

### Genotyping

The initial collection of wild males in September 2009 (*N*=31) as well as all flies from the adult phenotypes data set (*N*=436) were genotyped at the NERC Biomolecular Analysis Facility at the University of Sheffield, (Sheffield, UK) using previously identified (Wright *et al.*, 2004) and proven (Johns *et al.*, 2005) microsatellite loci. The eight X-linked loci were *ms71*, *ms125*, *ms244*, *ms395*, *mscrc2*, *ms54*, *ms106* and *ms167*. DNA was extracted by grinding each fly with a pestle and following a set extraction protocol: for each sample 48 μl of squishing buffer (25 mM NaCl, 1 mM EDTA, 10 mM Tris–Cl pH 8.2) and 2 μl Proteinase K (10 mg ml^−1^) was used, and incubated at 56 °C for 1.5 h, then treated with a heat shock at 90 °C for 5 min (Gloor *et al.*, 1993). PCR reactions were performed on a 2720 Thermal Cycler (Applied Biosystems, Woolston, UK) in 2 μl volumes, which consisted of 1 μl dried genomic DNA, 1 μl QIAGEN Multiplex PCR Mastermix (QIAGEN, Manchester, UK) and 1 μl Primer mix, with all primers at a 0.2 μM concentration, and using an oil drop on top to avoid evaporation. Primers for the microsatellites were taken from Wright *et al.* (2004) and had been arranged into multiplexes with the help of Multiplex Manager 1.0 (Holleley and Geerts, 2009). A touchdown PCR method was used. As such, the PCR profile had an initial denaturation stage of 15 min at 95 °C, followed by 35 cycles of 94 °C for 30 s, 63 °C for 90 s (reducing in temperature by 1 °C every cycle to 49 °C). This was followed by an elongation step of 30 min at 60 °C and an indefinite hold at 4 °C. Negative and positive controls were used during DNA extraction and PCR to ensure that contamination had not occurred. An ABI3730 Genetic Analyzer (Applied Biosystems) was used to visualise the microsatellites, with a LIZ500 size standard. GENEMAPPER 4.0 was used to assign microsatellite allele sizes. One microsatellite marker (*ms71*) did not amplify sufficiently in any of our data sets, and thus all results were produced using the remaining seven X-linked microsatellites.

### Statistical analysis—wild males

All males that contributed fewer than 10 offspring to the next generation were discarded from analyses. This cutoff was chosen as the theoretical minimum needed for a *χ*^2^-test is *N*=5 (the expected number of males and females) in each 2 × 2 cell (Cochran, 1952). The association of each X-linked microsatellite locus with the offspring sex ratio of each male was examined. The sex ratio was defined as the proportion of males (the number of male offspring divided by the total number of offspring). Each microsatellite locus was tested for association with sex ratio bias in a generalised linear model, assuming a binomial error structure. This assesses the number of male offspring in each brood after controlling for differences in brood size. Microsatellite size was assessed as a nominal variable, split into groups of 10 base pairs. An additional analysis of microsatellites with significant associations was done, splitting the microsatellite allele sizes into two groups (above and below the mean) and comparing these to meiotic drive. This analysis ensured that approximately equal sample sizes were present in each group. Holm–Bonferroni corrections for multiple comparisons were performed (Holm, 1979). A direct test of the relationship between male eyespan and offspring sex ratio was performed, using the same generalised linear model as above, testing the offspring sex ratio against thorax, absolute eyespan and relative eyespan.

### Statistical analysis—adult phenotypes

We examined the relationship between X-linked loci and a number of phenotypic traits. The relationship between trait size and allele size was calculated using a standard least squares generalised linear model. The allele size metric for each microsatellite locus was calculated using the proportion of alleles that each individual possessed that were greater than the population mean. As each female had a maximum of two alleles for each locus, the assigned values were 0, 0.5 or 1. Male genotype was coded as 0 or 1 depending on whether their single allele was greater than the population mean. This was compared with a number of traits: thorax (a proxy for body size), absolute eyespan, relative eyespan, testis size, accessory gland size and fecundity. Relative eyespan was calculated by including thorax in the model as a covariate to control for body size. The analysis was split by sex (male or female). As different sites will generally have different allele size frequencies, we used ‘site' as a covariate (random effect) to ensure the results reflected true associations with sex ratio bias and were not an artefact of the general site properties. The Holm–Bonferroni correction was applied (Holm, 1979), with each locus having five (4) tests for males (females).

The wild male data set suggested that *ms395* has a reliable association with sex ratio bias. We analysed whether *ms395* associated with different populations as well as population density. In order to calculate a single genotypic value for *ms395* for each individual, we categorised individuals as either having an allele size greater than 218 bp or not. This fitted with results showing that this locus had a bimodal distribution larger and smaller than 218bp. To examine associations of allele size with different populations we compared the allele size metric to ‘site' (different populations) using a likelihood ratio test. We also compared absolute allele sizes to population density using a generalised linear model with population sample size included as a covariate in order to remove effects related to sampling.

All statistical analysis was performed using JMP V. 10.0.0 (SAS Institute, Cary, NC, USA).

## Results

### Wild males

**Figure 2:**
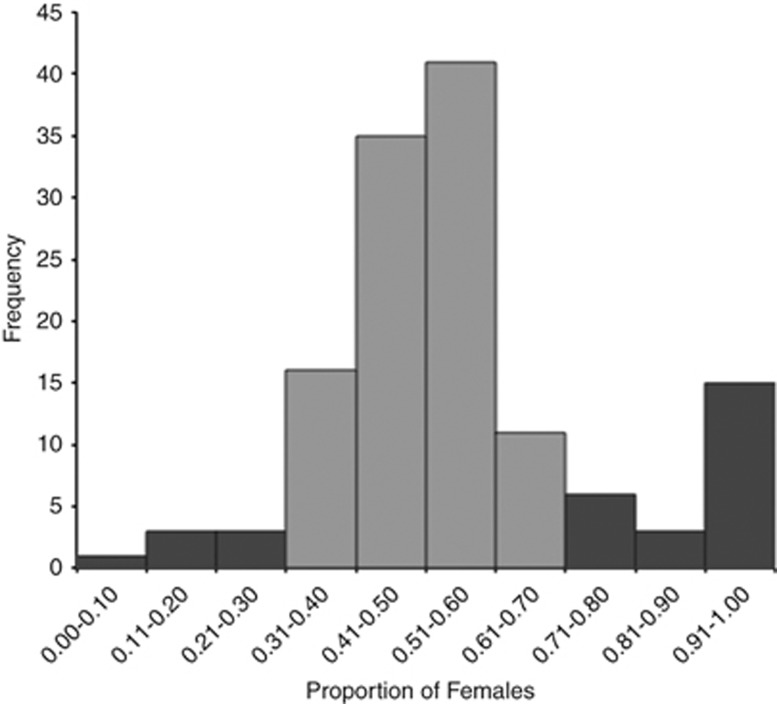
Frequency distribution of the proportion of female offspring in each brood for flies collected in 2009 and 2011 (*N*=134). Dark grey bars indicate sex ratios that differ significantly from 1:1.

Among the sample of flies taken in 2009, 22.6% produced significantly sex ratio biased broods (7/31). A similar pattern of 25.2% sex ratio distortion was found in 2011 (26/103) (Supplementary Table S1). Overall, most of the families with significant sex ratio distortion were female biased (25/134), but a few of which were male biased (8/134) (Figure 2; Supplementary Table S1).

**Figure 3:**
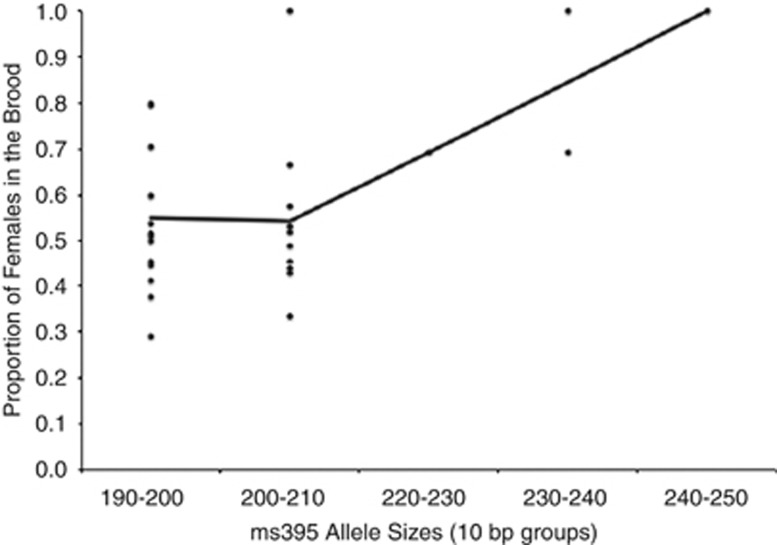
Association between sex ratio, given by the proportion of females in the brood, and *ms395* allele size given in 10 bp groupings. The line joins adjacent mean values. A significant relationship was found, with larger *ms395* alleles associated with more female-biased broods.

Locus *ms395* showed a significant relationship with sex ratio bias (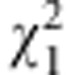
=44.7948, *N=*29, *P<*0.0001), with large *ms395* alleles being associated with more female-biased broods (Figure 3). Locus *ms54* also showed an association with sex ratio bias (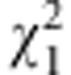
=7.5802, *N=*25, *P=*0.0226), again with large allele sizes being associated with more female-biased broods. None of the other loci showed a significant association with sex ratio bias (*mscrc2* 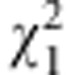
=3.3655, *N=*30, *P=*0.7618; *ms106* 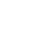
=0.7672, *N=*15, *P=*0.3811; *ms244* 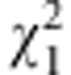
=2.4851, *N=*28, *P=*0.4780; *ms125* 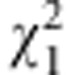
=3.5526, *N=*29, *P=*0.4699; *ms167* 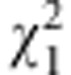
=0.2596, *N=*23, *P=*0.6104. After applying the Holm–Bonferroni correction, *ms395* remained significant, whereas *ms54* was rendered non-significant. When *ms395* allele sizes were split into two groups (above and below the mean (205 bp)) and compared with sex ratio bias, we also found a significant association (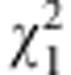
=23.3450, *N=*29, *P<*0.0001).

We found no relationship between sex ratio bias and body size (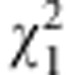
=1.3686, *N=*130, *P=*0.2421) or absolute eyespan (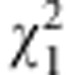
=1.2790, *N=*130, *P=*0.2581). We did, however, find a significant relationship between sex ratio bias and relative (male) eyespan (controlling for body size) with small relative eyespan males producing more female-biased broods (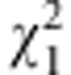
=6.9516, *N=*130, *P=*0.0084).

### Adult phenotypes and allele size

There was no relationship between body size and *ms395* allele size in either males (F_2,161.5_=0.6089, *P=*0.5452) or females (F_2,184.7_=1.4770, *P=*0.2310). Nor was there any relationship between absolute eyespan and *ms395* allele size in either sex (males: F_2,188_=2.0549, *P=*0.1310; females: F_2,179.7_=1.9029, *P=*0.1521). However, there was a significant negative association between male relative eyespan (after controlling for body size) and *ms395* allele size (F_2,182.8_=4.6991, *P=*0.0102), such that smaller eyespan males had larger *ms395* alleles. There was no equivalent relationship in females (F_2,183.1_=1.0540, *P=*0.3506). We also looked for associations between reproductive traits and *ms395* but found none in males with testis size (F_2,169.3_=1.0774, *P=*0.3428) or accessory gland size (F_2,168.9_=0.4284, *P=*0.6523) and none in females with fecundity (F_2,187.8_=0.0147, *P=*0.9854). Holm–Bonferroni corrections did not alter the significance of the relationship between relative eyespan and *ms395* (*P*<0.05).

The other six X-linked loci were also examined for associations with the phenotypic traits (Table 1). Several loci were again associated with male, but not female, relative eyespan (*ms54, ms244, mscrc2*) and there was an association with accessory gland size (*ms54*) and testis size (*mscrc2*).

**Figure 4:**
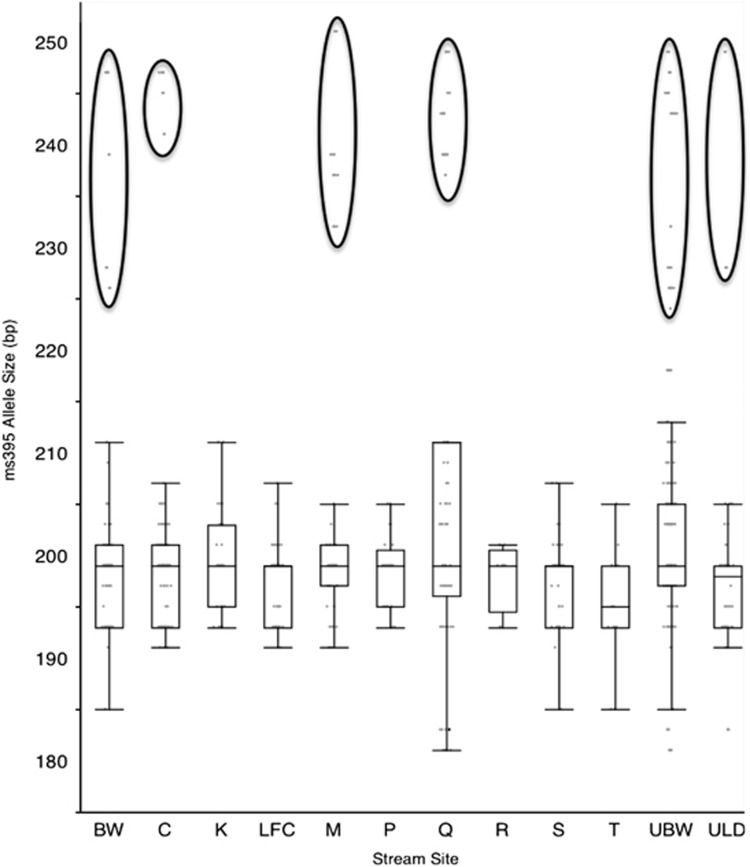
Box plot (Q1, median, Q3) of *ms395* allele sizes found at 12 sites along the Gombak valley (see Figure 1 for locations). Whiskers (Q1−1.5*interquartile range (IQR), Q3+1.5*IQR) show the spread of the allele sizes and outliers (mainly large alleles). Six of the 12 sites (circled) show the presence of large *ms395* alleles (>218 bp), whereas the other six sites show a complete absence of large alleles.

From the previous data set, we identified *ms395* as the only locus to show a reliable association with sex ratio bias. In order to investigate this further, we examined the frequency of *ms395* in different populations. We found a significant difference between sites in allele size at locus *ms395* (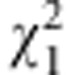
=36.7211, *N=*390, *P<*0.0001). When this is viewed graphically (Figure 4), it is clear that 6 of the 12 sites contain large *ms395* alleles (>218 bp) that are associated with meiotic drive, and in all cases the large alleles are outliers (using a box plot and whiskers). In addition, these sites represent geographically distinct populations along the valley (Figure 1). When we compared the population density of each site (flies per metre of sampled site) with the *ms395* alleles found in that site, controlling for sample size, we found a significant positive relationship, such that sites with large populations were associated with a high frequency of large *ms395* alleles (F_1582_=13.2839, *P=*0.0003).

## Discussion

We investigated meiotic drive in wild populations of the stalk-eyed fly, *T. dalmanni*. First, we examined the relationship between meiotic drive, measured as sex ratio distortion of progeny, and a number of X-linked microsatellite loci (Wright *et al.*, 2004; Johns *et al.*, 2005). Locus *ms395* showed a strong relationship with levels of meiotic drive. Large *ms395* alleles (>218 bp) were associated with female-biased broods. In addition, previous work in a laboratory population of *T. dalmanni* found that large *ms395* alleles were linked with meiotic drive (Johns *et al.*, 2005). It would be interesting to establish whether the specific association of ‘large' alleles of *ms395* and sex ratio distortion is due to some process that favours the accumulation of repeats in regions associated with meiotic drive. The same pattern is seen for the *Rsp* locus of the *SD* system in *D. melanogaster*, which has high repeat numbers in sensitive alleles (Larracuente and Presgraves, 2012). Typically, meiotic drive systems are found in areas of low recombination (Jaenike, 2001), but how this might predispose repeats to increases in number is unclear (Dion and Wilson, 2009). Locus *ms54* also showed an association with meiotic drive and this locus was also shown to be associated with meiotic drive in previous laboratory studies (Johns *et al.,* 2005). The relationship did not survive, however, after the Holm–Bonferroni correction was applied.

The laboratory study of *T. dalmanni* found that two other loci (*ms125* and *ms244*) were predictors of meiotic drive (Johns *et al.*, 2005). However, we found no association with *ms125* or *ms244*. The laboratory and wild populations were both collected from the same river catchment in Malaysia. However, differences could have built up in the laboratory population over time since collection, especially, as samples of the laboratory population were subjected to artificial selection (for relative male eyespan) and hence to random genetic drift. It is possible that low frequency microsatellite alleles that happened to be in linkage with the meiotic drive locus in the samples used for artificial selection spread to fixation by chance, and thus were identified as co-varying with meiotic drive. The wild populations used in this study were collected in 2008/9, whereas those that founded the laboratory population were collected in 1989 (Johns *et al.*, 2005). It is possible that the difference in our results in due to a rapid turnover of the drive complex in natural populations, which is supported by recent work that has provided evidence for the rapid evolution of the sex ratio complex, over only a few decades, in *D. simulans* (Bastide *et al.,* 2011).

Previous theoretical (Lande and Wilkinson, 1999) and experimental laboratory work (Wilkinson *et al.*, 1998; Johns *et al.*, 2005) has examined the hypothesis that male eyespan is linked to the presence or absence of the X^D^ chromosome. We complemented this work by examining wild-caught stalk-eyed flies in two independent data sets. We found that male eyespan correlated with meiotic drive directly in our wild male data set. In addition, we also found that male eyespan was correlated with microsatellite *ms395* size. Males with large allele sizes not only had female-biased sex ratios but also small relative eyespan, in line with the direction of results from our wild male data set. In contrast, there was no association of *ms395* allele size with the control trait, female eyespan, suggesting that linkage has specifically evolved between male eyespan and drive, which is consistent with previous findings from quantitative trait locus mapping (Johns *et al.*, 2005). The second microsatellite to correlate (before Holm–Bonferroni corrections) with meiotic drive (*ms54*) also associated with male eyespan. These results support the hypothesis that meiotic drive could be a factor in the evolution and maintenance of female mate choice for male eyespan size (Wilkinson *et al.*, 1998). Theory suggests that in order to maintain the linkage between male eyespan and meiotic drive, genes for both traits need to be contained within the same inversion (Lande and Wilkinson, 1999; Pomiankowski and Hurst, 1999). Two other X-linked microsatellites were associated with male eyespan but not with meiotic drive (*ms244* and *mscrc2*). This indicates that male eyespan is likely to be controlled by a number of different genes, an observation in line with previous work examining quantitative trait loci for eyespan in this species (Wolfenbarger and Wilkinson, 2001; Johns *et al.*, 2005). The most complete linkage map also places these microsatellites in close proximity (all within 20 cM) on the X chromosome, and thus it is possible that they are in linkage disequilibrium (Baker and Wilkinson, 2010), hence the close relationship with male eyespan.

We did not find any associations of *ms395* with male reproductive traits (accessory gland size and testis size), although we did find an association between *ms54* and accessory gland size. Accessory gland size is related to male mating rate (Baker *et al.*, 2003; Rogers *et al.*, 2005), and thus our results indicate that meiotic drive males may be constrained in their mating rate. This is in agreement with work by Wilkinson *et al.* (2006), who found that drive males produced fewer offspring than non-drive males and exhibited lower sperm precedence suggesting that there are costs of drive in terms of sperm number or competitive ability (Wilkinson *et al.*, 2006). They found no difference in the number of drive and non-drive males that produced offspring when mated multiply over a 24-h period, however, suggesting that the relationship between accessory gland size and meiotic drive may not be straightforward. We did not find any association of *ms395* (or any other locus) with female fecundity. In all of the analyses relating to associations between genotype and phenotypic traits, we controlled for general allelic variation between streams by adding stream as a covariate in every model. Because of the lack of detailed information on population structure in the valley, we cannot eliminate a potential role that population structure alone may have had on creating associations.

Before this study, there was little evidence for the existence and pattern of meiotic drive in the wild. Our results indicate that there is substantial variation in meiotic drive both within and between local wild populations. We found that half of the sites that were sampled from a single river valley did not exhibit alleles associated with meiotic drive (that is, contained no large *ms395* alleles >218 bp, Figure 4), whereas there were varying degrees of association with alleles associated with meiotic drive in the other sites. Migration between different sites is likely limited (S Cotton, unpublished data), suggesting that stochastic variation may build up at each locality. Differences in population density may explain the observed variation in meiotic drive. We found a significant correlation between density and the level of meiotic drive alleles observed. This was true even after controlling for sample size. There was no meiotic drive alleles in small populations, with levels of drive alleles increasing as population density increased. One possible explanation for this relationship is that if meiotic drive invades a small population, then that population would quickly become strongly female-biased and have a higher chance of going extinct (Hamilton, 1967; Jaenike, 2001). Selection is a weaker force in small populations (Crow and Kimura, 1970), so they are less likely to retain or evolve suppressors and thus less able to counter the spread of sex ratio distorting meiotic drive.

Wilkinson *et al.* (1998) proposed that female mate choice for large male eyespan may have evolved in the stalk-eyed fly as a form of ‘good genes' selection. This hypothesis was conceived following the finding in a laboratory experiment that the male sexual character (exaggerated eyespan) in stalk-eyed flies was associated with meiotic drive. This finding has not spurred further examination of the hypothesis, perhaps because the association between meiotic drive and eyespan could easily have arisen by chance, due to the laboratory-breeding regime used. Here we examined variation in meiotic drive, microsatellite markers and the associated sexual trait in wild populations of stalk-eyed flies. We found that two of the four microsatellite loci previously identified in the laboratory study were associated with meiotic drive, one (*ms395*) very strongly. We further confirmed, using two independent data sets, that there is a strong correlation between male eyespan and the microsatellite locus linked to drive. Our results constitute the first evidence from wild populations that the evolution of female mate choice for male eyespan is plausibly linked to a ‘good genes' hypothesis of avoiding prospective mates that harbour an X-linked meiotic drive chromosome.

## Data archiving

All data (genotypes and phenotypes) have been deposited in Dryad: doi:10.5061/dryad.jk2qr.

## Figures and Tables

**Table 1:** Relationships between X-linked microsatellite loci and phenotypic traits

*Locus*	*Sex*	*Body Size*			*Absolute eyespan*			*Relative eyespan*			*Testis size*			*Accessory gland size*			*Fecundity*		
		*F*	*d.f.*	P*-value*	*F*	*d.f.*	P*-value*	*F*	*d.f.*	P*-value*	*F*	*d.f.*	P*-value*	*F*	*d.f.*	P*-value*	*F*	*d.f.*	P*-value*
*ms54*	M	3.4844	1,201	0.0634	0.0349	1,83.9	0.8522	14.9213	1,195	**0.0002**	3.5872	1,139.7	0.0603	8.3223	1,84.72	**0.0050**			
*ms54*	F	1.7550	2,147.9	0.1765	3.0286	2,184.1	0.0508	1.9975	2,146.6	0.1393							0.1518	2,164.2	0.8593
*ms106*	M	0.9897	1,73.99	0.3231	0.0556	1,15.15	0.8167	3.7209	181	0.0572	0.0947	1,69.18	0.7592	0.5297	1,25.26	0.4734			
*ms106*	F	0.6653	1,54.22	0.4183	0.8992	1,19.55	0.3546	6.5939	1,33.55	**0.0149**							0.3299	1,52.81	0.5682
*ms125*	M	1.0822	1,210.1	0.2994	0.2420	1,201.7	0.6233	0.4469	1,206.8	0.5046	0.0201	1,195.4	0.8874	0.1169	1,193.3	0.8874			
*ms125*	F	0.4045	2,199	0.6679	0.8611	2,194.9	0.4243	1.7193	2,196.7	0.1819							1.1876	2,201.7	0.3071
*ms167*	M	0.0984	2,61.7	0.9064	0.1612	2,58.19	0.8515	0.0879	2,50.59	0.9160	2.6549	2,46.84	0.0809	0.0956	1,55.16	0.7584			
*ms167*	F	0.2357	2,78.34	0.7906	0.5474	2,81	0.5806	1.3935	2,77.11	0.2544							1.1244	2,82.53	0.3298
*ms244*	M	0.1667	2,33	0.8472	1.3315	2,32	0.2783	6.7569	2,28.64	**0.0039**	1.5034	2,29.22	0.2391	1.0959	2,24.39	0.3502			
*ms244*	F	4.3378	2,38	0.0201	2.0294	2,37.75	0.1455	0.1829	2,33.66	0.8336							1.0324	2,38.3	0.3658
*ms395*	M	0.6089	2,161.5	0.5452	2.0549	2,188	0.1310	4.6991	2,182.8	**0.0102**	1.0774	2,169.3	0.3428	0.4284	2,168.9	0.6523			
*ms395*	F	1.0581	2,189	0.3492	1.0782	2,188	0.3423	0.9660	2,187	0.3000							0.0147	2,187.8	0.9854
*mscrc2*	M	2.2662	1,211	0.1337	0.0753	1,168.2	0.7841	9.1645	1,204	**0.0028**	10.0399	1,197.5	**0.0018**	3.1427	1,187.8	0.0779			
*mscrc2*	F	1.0876	2,175.1	0.3393	3.1723	2,192.4	0.0441	2.5250	2,158	0.0833							0.3480	2,184.3	0.7680

Abbreviations: d.f., degree of freedom; F, female; M, male.

*P-*values in bold remained significant after the Holm–Bonferroni correction (*P*<0.05), those underlined were not significant after this correction (*P*>0.05).
