# Supplementary material for: Male eyespan size is associated with meiotic drive in wild stalk-eyed flies (Teleopsis dalmanni)
Source: Heredity (Edinb). 2014 Jan 8;112(4):363–9. doi: 10.1038/hdy.2013.131 (PMC3966131; doi:10.1038/hdy.2013.131)
Supplement: Supplementary Information [file hdy2013131x1.doc]

**Supplementary Material**

**Table S1: Offspring sex ratios of wild males.** Table showing raw offspring sex ratios for each wild male, along with chi square and *P* values. Significant (*P* < 0.05) values are highlighted in bold. Morphological data (eyespan and thorax) is also given.

| *Male ID* | *Eyespan* | *Thorax* | *Male offspring* | *Female offspring* | *Total offspring* | *Proportion of females* | *Expected* | *Chi Square* | *P Value* |
| --- | --- | --- | --- | --- | --- | --- | --- | --- | --- |
| BW2 | 7.9 | 2.19 | 10 | 39 | 49 | 0.7959 | 24.5 | 17.1633 | ***P* < 0.0001** |
| BW3 | 7.6 | 2.39 | 15 | 17 | 32 | 0.5313 | 16 | 0.1250 | *P* = 0.7237 |
| BW9 | 8.55 | 2.12 | 0 | 28 | 28 | 1.0000 | 14 | 28.0000 | ***P* < 0.0001** |
| BW10 | 5.52 | 1.78 | 13 | 14 | 27 | 0.5185 | 13.5 | 0.0370 | *P* = 0.8474 |
| C1 | 8.95 | 2.64 | 5 | 12 | 17 | 0.7059 | 8.5 | 2.8824 | *P* = 0.0896 |
| C8 | 5.3 | 2.05 | 6 | 5 | 11 | 0.4545 | 5.5 | 0.0909 | *P* = 0.7630 |
| C10 | 9.91 | 2.77 | 4 | 6 | 10 | 0.6000 | 5 | 0.4000 | *P* = 0.5271 |
| C14 | 7.11 | 2.04 | 4 | 9 | 13 | 0.6923 | 6.5 | 1.9231 | *P* = 0.1655 |
| C19 | 7.28 | 2.24 | 10 | 5 | 15 | 0.3333 | 7.5 | 1.6667 | *P* = 0.1967 |
| Q1 | 8.34 | 2.47 | 8 | 18 | 26 | 0.6923 | 13 | 3.8462 | ***P* = 0.0499** |
| Q2 | 8.9 | 2.55 | 13 | 9 | 22 | 0.4091 | 11 | 0.7273 | *P* = 0.3938 |
| Q4 | 5.2 | 1.63 | 24 | 23 | 47 | 0.4894 | 23.5 | 0.0213 | *P* = 0.8840 |
| Q6 | 7.05 | 2.21 | 9 | 7 | 16 | 0.4375 | 8 | 0.2500 | *P* = 0.6171 |
| Q7 | 3.78 | 1.52 | 10 | 6 | 16 | 0.3750 | 8 | 1.0000 | *P* = 0.3173 |
| Q11 | 5.53 | 1.96 | 3 | 12 | 15 | 0.8000 | 7.5 | 5.4000 | ***P* = 0.0201** |
| Q12 | 6.36 | 2.23 | 5 | 12 | 17 | 0.7059 | 8.5 | 2.8824 | *P* = 0.0896 |
| UBW1 | 7.27 | 2.27 | 24 | 24 | 48 | 0.5000 | 24 | 0.0000 | *P* = 1.0000 |
| UBW2 | 6.88 | 2.2 | 22 | 9 | 31 | 0.2903 | 15.5 | 5.4516 | ***P* = 0.0196** |
| UBW3 | 8.28 | 2.32 | 10 | 8 | 18 | 0.4444 | 9 | 0.2222 | *P* = 0.6374 |
| UBW5 | 9.28 | 2.69 | 12 | 13 | 25 | 0.5200 | 12.5 | 0.0400 | *P* = 0.8415 |
| UBW6 | 8.22 | 2.52 | 6 | 7 | 13 | 0.5385 | 6.5 | 0.0769 | *P* = 0.7815 |
| UBW11 | 10.1 | 2.37 | 5 | 10 | 15 | 0.6667 | 7.5 | 1.6667 | *P* = 0.1967 |
| UBW13 | 6.59 | 1.97 | 10 | 7 | 17 | 0.4118 | 8.5 | 0.5294 | *P* = 0.4669 |
| UBW14 | 9.31 | 2.47 | 22 | 23 | 45 | 0.5111 | 22.5 | 0.0222 | *P* = 0.8815 |
| UBW16 | 7.59 | 2.12 | 0 | 14 | 14 | 1.0000 | 7 | 14.0000 | ***P* = 0.0002** |
| UBW21 | 7.29 | 2.44 | 5 | 7 | 12 | 0.5833 | 6 | 0.3333 | *P* = 0.5637 |
| UBW22 | 7.66 | 2.23 | 17 | 23 | 40 | 0.5750 | 20 | 0.9000 | *P* = 0.3428 |
| UBW23 | 6.09 | 2.01 | 16 | 12 | 28 | 0.4286 | 14 | 0.5714 | *P* = 0.4497 |
| UBW27 | 6.72 | 2.19 | 0 | 16 | 16 | 1.0000 | 8 | 16.0000 | ***P* < 0.0001** |
| ULD2 | 7.97 | 2.16 | 12 | 10 | 22 | 0.4545 | 11 | 0.1818 | *P* = 0.6698 |
| ULD14 | 9.31 | 2.47 | 4 | 6 | 10 | 0.6000 | 5 | 0.4000 | *P* = 0.5271 |
| 5 | 6.53 | 1.66 | 3 | 17 | 20 | 0.8500 | 10 | 9.8000 | ***P* = 0.0017** |
| 7 | 8.51 | 2.05 | 58 | 48 | 106 | 0.4528 | 53 | 0.9434 | *P* = 0.3314 |
| 8 | 6.91 | 1.83 | 0 | 32 | 32 | 1.0000 | 16 | 32.0000 | ***P* < 0.0001** |
| 23 | 6.67 | 1.46 | 23 | 21 | 44 | 0.4773 | 22 | 0.0909 | *P* = 0.7630 |
| 10 | 7.22 | 1.38 | 34 | 23 | 57 | 0.4035 | 28.5 | 2.1228 | *P* = 0.1451 |
| 11 | 6.14 | 1.76 | 16 | 17 | 33 | 0.5152 | 16.5 | 0.0303 | *P* = 0.8618 |
| 15 | 7.76 | 1.63 | 43 | 36 | 79 | 0.4557 | 39.5 | 0.6203 | *P* = 0.4310 |
| 22 | 6.18 | 1.97 | 18 | 19 | 37 | 0.5135 | 18.5 | 0.0270 | *P* = 0.8694 |
| 26 |  |  | 1 | 9 | 10 | 0.9000 | 5 | 6.4000 | ***P* = 0.0114** |
| 29 | 7.72 | 1.84 | 7 | 3 | 10 | 0.3000 | 5 | 1.6000 | *P* = 0.2059 |
| 31 | 5.96 | 1.69 | 34 | 44 | 78 | 0.5641 | 39 | 1.2821 | *P* = 0.2575 |
| 35 | 5.47 | 1.75 | 7 | 91 | 98 | 0.9286 | 49 | 72.0000 | ***P* < 0.0001** |
| 39 | 6.13 | 1.4 | 44 | 41 | 85 | 0.4824 | 42.5 | 0.1059 | *P* = 0.7449 |
| 40 | 5.79 | 1.6 | 0 | 113 | 113 | 1.0000 | 56.5 | 113.0000 | ***P* < 0.0001** |
| 41 | 5.07 | 1.64 | 52 | 56 | 108 | 0.5185 | 54 | 0.1481 | *P* = 0.7003 |
| 42 | 9.19 | 2.54 | 19 | 4 | 23 | 0.1739 | 11.5 | 9.7826 | ***P* = 0.0018** |
| 43 | 5.94 | 1.95 | 22 | 38 | 60 | 0.6333 | 30 | 4.2667 | ***P* = 0.0389** |
| 44 | 7.51 | 2.04 | 45 | 76 | 121 | 0.6281 | 60.5 | 7.9421 | ***P* = 0.0048** |
| 45 | 7.46 | 1.72 | 3 | 98 | 101 | 0.9703 | 50.5 | 89.3564 | ***P* < 0.0001** |
| 46 | 7.99 | 2.26 | 2 | 71 | 73 | 0.9726 | 36.5 | 65.2192 | ***P* < 0.0001** |
| 47 | 7.32 | 1.94 | 45 | 57 | 102 | 0.5588 | 51 | 1.4118 | *P* = 0.2348 |
| 48 | 6.19 | 1.8 | 8 | 56 | 64 | 0.8750 | 32 | 36.0000 | ***P* < 0.0001** |
| 49 | 6.83 | 1.45 | 0 | 84 | 84 | 1.0000 | 42 | 84.0000 | ***P* < 0.0001** |
| 51 | 9.19 | 2.14 | 44 | 55 | 99 | 0.5556 | 49.5 | 1.2222 | *P* = 0.2689 |
| 52 | 7.17 | 1.98 | 54 | 36 | 90 | 0.4000 | 45 | 3.6000 | *P* = 0.0578 |
| 53 | 7.41 | 1.97 | 14 | 9 | 23 | 0.3913 | 11.5 | 1.0870 | *P* = 0.2971 |
| 54 | 7.56 | 2.04 | 27 | 35 | 62 | 0.5645 | 31 | 1.0323 | *P* = 0.3096 |
| 55 | 6.63 | 2.15 | 48 | 51 | 99 | 0.5152 | 49.5 | 0.0909 | *P* = 0.7630 |
| 56 | 8.86 | 2.34 | 25 | 22 | 47 | 0.4681 | 23.5 | 0.1915 | *P* = 0.6617 |
| 58 | 6.43 | 1.83 | 14 | 10 | 24 | 0.4167 | 12 | 0.6667 | *P* = 0.4142 |
| 59 | 6.71 | 1.85 | 49 | 45 | 94 | 0.4787 | 47 | 0.1702 | *P* = 0.6799 |
| 62 | 9.08 | 2.21 | 21 | 30 | 51 | 0.5882 | 25.5 | 1.5882 | *P* = 0.2076 |
| 63 | 7.52 | 1.93 | 17 | 19 | 36 | 0.5278 | 18 | 0.1111 | *P* = 0.7389 |
| 64 | 7.91 | 2.25 | 12 | 15 | 27 | 0.5556 | 13.5 | 0.3333 | *P* = 0.5637 |
| 65 | 6.1 | 1.78 | 20 | 12 | 32 | 0.3750 | 16 | 2.0000 | *P* = 0.1573 |
| 66 | 6.79 | 1.53 | 19 | 13 | 32 | 0.4063 | 16 | 1.1250 | *P* = 0.2888 |
| 67 | 9.17 | 2.41 | 11 | 2 | 13 | 0.1538 | 6.5 | 6.2308 | ***P* = 0.0126** |
| 68 | 8.86 | 2.46 | 39 | 46 | 85 | 0.5412 | 42.5 | 0.5765 | *P* = 0.4477 |
| 69 | 7.74 | 1.99 | 27 | 26 | 53 | 0.4906 | 26.5 | 0.0189 | *P* = 0.8907 |
| 70 | 7.86 | 2.19 | 11 | 10 | 21 | 0.4762 | 10.5 | 0.0476 | *P* = 0.8273 |
| 71 | 8.69 | 2.1 | 16 | 23 | 39 | 0.5897 | 19.5 | 1.2564 | *P* = 0.2623 |
| 72 | 6.26 | 2.02 | 35 | 44 | 79 | 0.5570 | 39.5 | 1.0253 | *P* = 0.3113 |
| 74 | 8.08 | 1.98 | 12 | 17 | 29 | 0.5862 | 14.5 | 0.8621 | *P* = 0.3532 |
| 77 | 8.69 | 2.96 | 15 | 10 | 25 | 0.4000 | 12.5 | 1.0000 | *P* = 0.3173 |
| 78 | 9.2 | 2.09 | 17 | 15 | 32 | 0.4688 | 16 | 0.1250 | *P* = 0.7237 |
| 79 | 7.15 | 2.17 | 15 | 22 | 37 | 0.5946 | 18.5 | 1.3243 | *P* = 0.2498 |
| 80 | 8.3 | 1.74 | 40 | 40 | 80 | 0.5000 | 40 | 0.0000 | *P* = 1.0000 |
| 81 | 8.8 | 1.87 | 27 | 38 | 65 | 0.5846 | 32.5 | 1.8615 | *P* = 0.1724 |
| 82 | 8.23 | 2.08 | 19 | 21 | 40 | 0.5250 | 20 | 0.1000 | *P* = 0.7518 |
| 85 | 7.92 | 2.26 | 15 | 11 | 26 | 0.4231 | 13 | 0.6154 | *P* = 0.4328 |
| 87 | 7.61 | 1.82 | 5 | 5 | 10 | 0.5000 | 5 | 0.0000 | *P* = 1.0000 |
| 88 | 7.5 | 1.59 | 17 | 22 | 39 | 0.5641 | 19.5 | 0.6410 | *P* = 0.4233 |
| 89 | 9.1 | 2.38 | 45 | 64 | 109 | 0.5872 | 54.5 | 3.3119 | *P* = 0.0688 |
| 90 | 6.81 | 1.72 | 27 | 25 | 52 | 0.4808 | 26 | 0.0769 | *P* = 0.7815 |
| 91 | 7.15 | 2.17 | 36 | 21 | 57 | 0.3684 | 28.5 | 3.9474 | ***P* = 0.0469** |
| 92 | 6.13 | 1.88 | 17 | 20 | 37 | 0.5405 | 18.5 | 0.2432 | *P* = 0.6219 |
| 93 | 7.65 | 2.06 | 12 | 17 | 29 | 0.5862 | 14.5 | 0.8621 | *P* = 0.3532 |
| 94 | 7.35 | 2.14 | 20 | 29 | 49 | 0.5918 | 24.5 | 1.6531 | *P* = 0.1985 |
| 95 | 7.59 | 2.09 | 7 | 10 | 17 | 0.5882 | 8.5 | 0.5294 | *P* = 0.4669 |
| 96 | 6.79 | 1.8 | 20 | 18 | 38 | 0.4737 | 19 | 0.1053 | *P* = 0.7456 |
| 97 | 7.38 | 1.67 | 16 | 19 | 35 | 0.5429 | 17.5 | 0.2571 | *P* = 0.6121 |
| 99 |  |  | 0 | 40 | 40 | 1.0000 | 20 | 40.0000 | ***P* < 0.0001** |
| LD10 | 6.4 | 1.26 | 68 | 61 | 129 | 0.4729 | 64.5 | 0.3798 | *P* = 0.5377 |
| LD11 | 7.58 | 1.41 | 28 | 34 | 62 | 0.5484 | 31 | 0.5806 | *P* = 0.4461 |
| LD14 | 6.19 | 1.13 | 13 | 23 | 36 | 0.6389 | 18 | 2.7778 | *P* = 0.0956 |
| LD15 | 5.36 | 1.09 | 3 | 8 | 11 | 0.7273 | 5.5 | 2.2727 | *P* = 0.1317 |
| LD16 | 7.28 | 1.24 | 31 | 23 | 54 | 0.4259 | 27 | 1.1852 | *P* = 0.2763 |
| LD17 | 5.88 | 1.18 | 7 | 4 | 11 | 0.3636 | 5.5 | 0.8182 | *P* = 0.3657 |
| LD18 | 5.07 | 1.12 | 14 | 20 | 34 | 0.5882 | 17 | 1.0588 | *P* = 0.3035 |
| LD19 | 6.36 | 1.21 | 57 | 48 | 105 | 0.4571 | 52.5 | 0.7714 | *P* = 0.3798 |
| LD20 | 6.63 | 1.31 | 40 | 40 | 80 | 0.5000 | 40 | 0.0000 | *P* = 1.0000 |
| LD22 | 4.95 | 1.19 | 6 | 4 | 10 | 0.4000 | 5 | 0.4000 | *P* = 0.3800 |
| LD23 | 8.7 | 1.44 | 26 | 22 | 48 | 0.4583 | 24 | 0.3333 | *P* = 0.5637 |
| LD24 | 4.97 | 0.97 | 24 | 27 | 51 | 0.5294 | 25.5 | 0.1765 | *P* = 0.6744 |
| LD29 | 7.18 | 1.39 | 28 | 17 | 45 | 0.3778 | 22.5 | 2.6889 | *P* = 0.1011 |
| LD30 |  |  | 39 | 35 | 74 | 0.4730 | 37 | 0.2162 | *P* = 0.6419 |
| LD33 | 6.26 | 1.27 | 41 | 26 | 67 | 0.3881 | 33.5 | 3.3582 | *P* = 0.0669 |
| LD34 | 6.7 | 1.2 | 20 | 17 | 37 | 0.4595 | 18.5 | 0.2432 | *P =* 0.6219 |
| LD36 | 5.43 | 1.15 | 31 | 37 | 68 | 0.5441 | 34 | 0.5294 | *P =* 0.4669 |
| LD37 | 4.03 | 0.95 | 14 | 19 | 33 | 0.5758 | 16.5 | 0.7576 | *P =* 0.3841 |
| LD1 | 6.64 | 1.41 | 1 | 12 | 13 | 0.9231 | 6.5 | 9.3077 | ***P =* 0.0023** |
| LD6 | 7.26 | 1.24 | 14 | 46 | 60 | 0.7667 | 30 | 17.0667 | ***P* < 0.0001** |
| LD8 | 6.14 | 1.27 | 39 | 21 | 60 | 0.3500 | 30 | 5.4000 | ***P* = 0.0201** |
| LD13 | 8.59 | 1.59 | 27 | 44 | 71 | 0.6197 | 35.5 | 4.0704 | ***P* = 0.0436** |
| LD28 | 5.88 | 1.15 | 22 | 9 | 31 | 0.2903 | 15.5 | 5.4516 | ***P* = 0.0196** |
| LD31 |  |  | 5 | 54 | 59 | 0.9153 | 29.5 | 40.6949 | ***P* < 0.0001** |
| LD32 | 8.59 | 1.82 | 0 | 54 | 54 | 1.0000 | 27 | 54.0000 | ***P* < 0.0001** |
| LD35 | 7.25 | 1.35 | 14 | 0 | 14 | 0.0000 | 7 | 14.0000 | ***P* = 0.0002** |
| BW11 | 4.21 | 1.03 | 30 | 40 | 70 | 0.5714 | 35 | 1.4286 | *P* = 0.2320 |
| BW13 | 5.89 | 1.16 | 0 | 29 | 29 | 1.0000 | 14.5 | 29.0000 | ***P* < 0.0001** |
| BW15 | 7.13 | 1.48 | 23 | 12 | 35 | 0.3429 | 17.5 | 3.4571 | *P* = 0.0630 |
| BW18 | 4.78 | 0.99 | 71 | 78 | 149 | 0.5235 | 74.5 | 0.3289 | *P* = 0.5663 |
| M1 | 6.43 | 1.21 | 11 | 10 | 21 | 0.4762 | 10.5 | 0.0476 | *P* = 0.8273 |
| Q12 | 5.82 | 1.14 | 0 | 17 | 17 | 1.0000 | 8.5 | 17.0000 | ***P* < 0.0001** |
| Q13 | 3.82 | 0.83 | 4 | 8 | 12 | 0.6667 | 6 | 1.3333 | *P* = 0.2482 |
| Q15 | 4.36 | 1.12 | 26 | 16 | 42 | 0.3810 | 21 | 2.3810 | *P* = 0.1228 |
| Q18 | 5.74 | 1.13 | 5 | 8 | 13 | 0.6154 | 6.5 | 0.6923 | *P* = 0.4054 |
| Q19 | 7.36 | 1.3 | 6 | 4 | 10 | 0.4000 | 5 | 0.4000 | *P* = 0.5271 |
| R13 | 6 | 1.24 | 4 | 7 | 11 | 0.6364 | 5.5 | 0.8182 | *P* = 0.3657 |
| R4 | 9.98 | 1.71 | 9 | 2 | 11 | 0.1818 | 5.5 | 4.4545 | ***P* = 0.0348** |
| R7 | 7.85 | 1.58 | 57 | 38 | 95 | 0.4000 | 47.5 | 3.8000 | *P* = 0.0513 |
| W1 | 5.07 | 1.19 | 3 | 7 | 10 | 0.7000 | 5 | 1.6000 | *P* = 0.2059 |
| W3 | 6.31 | 1.23 | 10 | 8 | 18 | 0.4444 | 9 | 0.2222 | *P* = 0.6374 |
